# Foretell Ovary: A Blood-Based Ensemble Algorithm Integrating Tumor-Educated Platelet RNA and Routine Hematology for Non-Invasive Ovarian Cancer Screening

**DOI:** 10.3390/cancers18162629

**Published:** 2026-08-14

**Authors:** Eunyong Ahn, Se Ik Kim, Sarah Kim, Hyunjung Kim, Hee-yeon Kim, Sungmin Park, Eun Ji Song, Hayoon Kwon, Kyung-Ah Hwang, Yong Sang Song, Jae-Hoon Kim, TaeJin Ahn

**Affiliations:** 1 Foretell My Health, Inc., Pohang 37554, Republic of Koreataejin.ahn@foretell.bio (T.A.); 2 Department of Obstetrics and Gynecology, Seoul National University Hospital, Seoul 03080, Republic of Korea; seikkim1@snu.ac.kr; 3 Department of Genomic Strategy, Samkwang Labtree, Seoul 06740, Republic of Korea; 4 Department of Obstetrics and Gynecology, Myongji Hospital, Goyang 10475, Republic of Korea; 5 Department of Obstetrics and Gynecology, Gangnam Severance Hospital, Yonsei University College of Medicine, Seoul 06273, Republic of Korea; 6 Department of Life Science, Handong Global University, Pohang 37554, Republic of Korea

**Keywords:** ovarian cancer, liquid biopsy, tumor-educated platelets, platelet RNA, complete blood count, ensemble machine learning, early detection, non-invasive screening, CA-125

## Abstract

**Background:** Ovarian cancer is the most lethal gynecologic malignancy, and CA-125 has limited early-stage sensitivity. We developed and evaluated Foretell Ovary, an ensemble machine-learning classifier that integrates tumor-educated-platelet RNA markers and routine hematologic parameters for non-invasive ovarian cancer detection, and compared it head-to-head with CA-125. **Methods:** A multi-site prospective cohort study enrolled 460 South Korean participants (67 ovarian cancer, 15 borderline ovarian tumors, 197 benign gynecological conditions, and 181 asymptomatic controls) at six clinical sites between September 2024 and March 2026. The cohort was split into training (*n* = 279) and independent test (*n* = 181) sets under a pre-specified ACTB cycle threshold ≤ 28 per-protocol rule (test *n* = 169). The classifier blended a random forest and two calibrated logistic-regression sub-models built on eight platelet RNA markers, 24 complete blood count parameters, and 30 marker-by-hematology combination features. Performance was assessed at a pre-frozen cutoff of 0.44 and compared against CA-125 (35 U/mL cutoff) in a matched subset (*n* = 100). **Results:** Foretell Ovary achieved an area under the receiver operating characteristic curve (AUC) of 0.923, sensitivity of 87.5% (100.0% for Stage II–IV), specificity of 89.0%, and negative predictive value of 97.7% in the test set. In the matched subset, Foretell Ovary outperformed CA-125 on every metric (AUC 0.839 vs. 0.723; sensitivity 85.0% vs. 65.0%), with the largest gap in pre-menopausal participants (+25 percentage points in sensitivity). A three-tier risk-grading scheme retained no ovarian cancer in the low-risk grade. **Conclusions:** Foretell Ovary offers more accurate non-invasive detection of ovarian cancer than CA-125, particularly in pre-menopausal women, offering the flexibility to be used alone or combined with CA-125. Prospective validation studies are warranted.

## Simple Summary

Ovarian cancer is often diagnosed at an advanced stage, and the widely used blood test CA-125 misses many early-stage cases and gives false positives in younger women. We developed Foretell Ovary, a new blood test that measures small amounts of RNA carried by platelets—a blood component that responds to signals from tumors—and combines those measurements with a routine blood count. In 460 people (including 67 with ovarian cancer and 15 with borderline tumors) across six hospitals in South Korea, our test correctly identified 87.5% of women who had ovarian cancer and correctly cleared 89.0% of women who did not, outperforming CA-125 especially in younger, pre-menopausal women. If confirmed in larger studies, this test could help detect ovarian cancer earlier and reduce unnecessary follow-up procedures.

## 1. Introduction

Ovarian cancer remains the most lethal gynecologic malignancy worldwide, accounting for an estimated 324,603 new cases and 206,956 deaths in 2022 [1]. In the United States alone, approximately 19,680 new diagnoses and 12,740 deaths were projected for 2024, placing ovarian cancer fifth among cancer-related deaths in women [2]. The disproportionate mortality burden of ovarian cancer relative to its incidence reflects a fundamental diagnostic challenge: the disease is typically asymptomatic in its early stages, and over 70% of patients present with advanced-stage (FIGO Stage III–IV) disease at the time of diagnosis [1,3]. The five-year overall survival rate for Stage III–IV ovarian cancer is approximately 20–40%, in stark contrast to over 90% for Stage I, underscoring the critical importance of early detection in improving patient outcomes [3].

Current clinical practice lacks an effective population-level screening strategy for ovarian cancer. The serum biomarker CA-125, introduced in 1983 [4], remains the most widely used ovarian cancer marker over four decades later, but exhibits sensitivity of only 50–60% for Stage I disease [3,5], is frequently elevated in benign gynecological conditions and inflammatory states, and performs particularly poorly in pre-menopausal women [5]. The UK Collaborative Trial of Ovarian Cancer Screening (*n* = 202,638) demonstrated that neither annual CA-125 monitoring nor transvaginal ultrasound significantly reduced ovarian cancer mortality [5]. Multi-biomarker algorithms such as the Risk of Ovarian Malignancy Algorithm (CA-125 + human epididymis protein 4) modestly improve specificity for benign-versus-malignant discrimination in pelvic-mass workups [6], but the fundamental limitation of early-stage sensitivity remains unresolved.

Among liquid-biopsy substrates, platelets are uniquely positioned to capture systemic tumor signals. Although anucleate, platelets inherit a diverse RNA cargo from megakaryocyte precursors and retain a spliceosome capable of signal-dependent pre-mRNA splicing upon activation [7,8]. Tumor-derived signals reprogram platelet RNA through direct exosomal transfer [9] and cytokine-mediated reprogramming of megakaryocytes [10], producing a distinctive tumor-educated-platelet transcriptomic fingerprint accessible from a standard blood draw. Best et al. (2015) achieved 96% accuracy across six cancer types (*n* = 283) [11]; In ’t Veld et al. (2022) extended this to 18 cancer types in 2351 individuals with 99% specificity [12]. For ovarian cancer, Gao et al. (2023) reported AUC 0.918 (*n* = 761; 0.922 with CA-125) [13], and Ahn et al. (2025) reported negative predictive value exceeding 99% using an isoform-splicing ratio approach [14].

Beyond platelet RNA reprogramming, ovarian cancer induces systemic perturbations captured by routine complete-blood-count indices such as paraneoplastic thrombocytosis [15], the systemic immune-inflammation index [16], neutrophil-to-lymphocyte ratio [17], and platelet-to-lymphocyte ratio [18]. Their diagnostic potential in combination with platelet RNA profiling has not been systematically exploited. Here, we present Foretell Ovary, a blood-based ensemble algorithm that integrates platelet RNA with routine hematological parameters, and compare it head-to-head with CA-125 in a matched subset.

## 2. Materials and Methods

### 2.1. Study Population and Design

This was a multi-site prospective cohort study at six enrollment sites in South Korea between September 2024 and March 2026. Sites are anonymized as Site 1 through Site 6; Site 2 is the asymptomatic-control enrollment cohort under a single Handong Global University Institutional Review Board approval. Eligible participants were adults aged ≥ 19 years compatible with one of three categories: ovarian cancer (confirmed histopathologically and staged per FIGO 2014), benign gynecological conditions (confirmed by imaging and pathology where available), or asymptomatic control (recruited from general health-screening settings). Borderline ovarian tumors were enrolled separately, not used for model training, and evaluated only as a reference subgroup. Participants with prior malignancy, active infection, or recent major surgery were excluded. The cohort was partitioned into training (*n* = 279) and independent test (*n* = 181) sets using a single pre-specified allocation, preferentially assigning participants with concurrent CA-125 measurements to the test set.

### 2.2. Blood Collection, Transport and Hematology

Peripheral blood was drawn into EDTA tubes (BD Vacutainer^®^, Franklin Lakes, NJ, USA, cat. 367525). Complete blood counts were acquired on the same automated hematology analyzer (Sysmex XP-300, Kobe, Japan) at each site. Samples were maintained at 4 °C, transported within 24 h, and processed within 48 h of draw using a differential centrifugation protocol. All blood samples were handled under Biosafety Level 2 (BSL-2) precautions with universal blood/body fluid precautions in accordance with the Korea Disease Control and Prevention Agency (KDCA) Laboratory Biosafety Management Manual.

### 2.3. RNA Extraction and Reverse Transcription

Platelet-rich plasma was isolated by differential centrifugation and total RNA was extracted (Thermo Fisher Scientific, Waltham, MA, USA, cat. A27828). RNA concentration and integrity were assessed by spectrophotometry (Implen NanoPhotometer, GmbH, Munich, Germany) and automated electrophoresis (Bioanalyzer, Santa Clara, CA, USA). Reverse transcription to complementary DNA used a commercial kit (Takara Bio, Kusatsu, Shiga, Japan, cat. RR047A) with random hexamer priming per manufacturer instructions.

### 2.4. RT-qPCR and Marker Panel

Eight platelet RNA markers were quantified by reverse-transcription quantitative PCR (RT-qPCR): IGFBP2-a, IGFBP2-b, IL1R2-a, IL1R2-b, LGALS3BP-a, WHAMMP3-a, LOC100420587-a, and FCGR2A-b. These markers were nominated from a prior platelet RNA-sequencing discovery program, prioritized by differential expression versus asymptomatic controls and versus benign gynecological conditions, fold-change magnitude, and Mann–Whitney U test significance. Candidate selection followed a staged assay-development process. A total of 112 differentially expressed transcripts identified from the discovery RNA-sequencing dataset were advanced for RT-qPCR primer design. Of these, 42 passed primer stability testing, 27 subsequently passed probe stability testing, and 8 ultimately passed reference-standard-based stability testing to form the final locked marker panel. No additional candidates satisfied all predefined assay performance criteria. Thus, the final panel comprised all markers that successfully met the predefined assay qualification criteria rather than an arbitrarily selected number. Two amplicon designs per transcript (-a, -b) targeting different exonic regions were retained to hedge against primer-specific efficiency variation. ACTB was used as the internal reference gene; primer and probe sequences are provided in the Supplementary Materials. Amplifications were performed on a real-time PCR system (CFX Opus 96 Real-Time PCR System, Bio-Rad Laboratories, Hercules, CA, USA, Cat. 12011319) in technical duplicate.

### 2.5. Quality Control and Per-Protocol Rule

Samples with ACTB cycle threshold (Cq) ≤ 28 were considered to have passed analytical quality control. The threshold was selected because it fell within a clear, unoccupied interval in the observed ACTB Cq distribution. Across all 460 participants, the highest ACTB Cq value among quality-control-passing samples was 24.1, whereas the lowest value among quality-control-failing samples was 29.2, with no observations between these values. Thus, any threshold within this approximately five-cycle interval would have yielded the same exclusion set. Six participants, all with benign gynecological conditions in the independent test set, had ACTB Cq > 28 and were excluded from the primary per-protocol analyses. Samples with ACTB Cq > 28 were reported as “test uninformative—repeat blood draw recommended”. Sensitivity analyses including these samples are provided in the Supplementary Materials, and an intent-to-test reference is additionally reported for the CA-125 matched comparison.

### 2.6. Feature Engineering and Ensemble Model

For each sample, ΔCq was computed as marker Cq minus ACTB Cq for within-sample normalization. Thirty-three raw features (eight RNA ΔCq plus 25 hematology parameters including derived indices systemic immune-inflammation index, neutrophil-to-lymphocyte ratio, platelet-to-lymphocyte ratio, and age) were generated, together with 4465 candidate combination features (pairwise and higher-order, up to four-way, arithmetic combinations—sum, difference, product, and ratio—among RNA ΔCq and hematology features). To select a stable subset for the combination-feature sub-model, all 4465 candidates were independently re-ranked across 15 resampling iterations (repeated stratified 5-fold cross-validation, 3 repeats) on the training set after class balancing with the Synthetic Minority Over-sampling Technique (SMOTE), using each feature’s univariate partial area under the receiver operating characteristic curve (restricted to ≤15% false-positive rate, evaluated in both feature directions); in each iteration, the top 60 of the 4465 candidates by this score were flagged. Across the 15 iterations, 100 distinct features were flagged in at least 20% (≥3) of iterations and were designated stable; the 30 most frequently flagged among these 100 were retained as the final combination-feature set.

Foretell Ovary is an ensemble of three sub-models: a random forest on the 33 raw features (weight 0.60), a calibrated logistic regression on the same raw features (0.28), and a calibrated logistic regression on the 30 stable combination features (0.12). These weights were established through an exploratory grid search across hundreds to thousands of candidate sub-model and weighting combinations. In practical terms, the three sub-models can be understood as independent readers evaluating each case from different perspectives—a pattern-based reader (random forest), a linear-trend reader on individual markers (raw-feature logistic regression), and a linear-trend reader on marker-hematology interactions (combination-feature logistic regression)—whose opinions are combined by a fixed, pre-specified weighting rather than a simple majority vote. To evaluate whether this combination provides benefit beyond any single sub-model, each component was assessed independently at the same deployed cutoff (Table S1). The random forest sub-model achieved a comparable AUC to the ensemble (0.926 vs. 0.923) but with markedly lower sensitivity (66.7% vs. 87.5%), missing 8 of 24 cancers that the ensemble detected; the raw-feature logistic regression matched the ensemble’s sensitivity but with lower specificity (82.1% vs. 89.0%). The ensemble achieved the highest balanced accuracy of any configuration (88.2%), a 3.4-percentage-point improvement over the best-performing single sub-model (raw-feature logistic regression, 84.8%), by combining the random forest’s specificity with the raw-feature logistic regression’s sensitivity. Class imbalance was addressed with SMOTE. Random-forest hyperparameters were selected by RandomizedSearchCV with stratified partial-AUC optimization, and sub-models were calibrated with sigmoid calibration using 5-fold cross-validation on the training set only. The classification cutoff of 0.44 was selected on the training set as the operating point maximizing the Youden index subject to a 90% specificity bound, and was frozen prior to any test-set evaluation.

### 2.7. Three-Tier Risk Grading

Raw scores were transformed to a 0–100 point scale and stratified into low risk (raw < 0.200; 0–60 points), intermediate risk (0.200 ≤ raw < 0.44; 60–80 points), and high risk (raw ≥ 0.44; 80–100 points). The low-risk threshold was selected on the training set as the highest value at which no training-set ovarian cancer sample (borderline tumors excluded) was classified as low risk, with a safety margin of ≥0.005 (final margin +0.008). Both thresholds were frozen on the training set prior to test-set evaluation.

### 2.8. CA-125 Subgroup Analysis

Because serum CA-125 measurements were not available for all enrolled participants, the head-to-head analysis was restricted to clinical samples from participants in the ovarian cancer or benign gynecological categories for whom both CA-125 measurements and Foretell Ovary scores were available. This was a paired-measurement comparison rather than a demographic or case–control matched analysis. Borderline tumors and nonclinical control samples were excluded. The resulting CA-125 comparison subset comprised 105 participants (ovarian cancer, *n* = 20; benign, *n* = 85). Of these, five benign samples failed the ACTB quality-control criterion; therefore, the per-protocol analysis included 100 participants (ovarian cancer, *n* = 20; benign, *n* = 80), while results for all 105 participants were reported in parallel as an intent-to-test analysis. The standard clinical cutoff of 35 U/mL was applied for CA-125. Subgroup analyses by menopausal status and two logical combination strategies (OR and AND) were evaluated. A stage-stratified subgroup analysis was additionally performed to evaluate Stage I performance specifically (Supplementary Table S2).

### 2.9. Statistical Analysis

Group comparisons used the Kruskal–Wallis test across three groups or the Mann–Whitney U test for pairwise comparisons (two-sided); effect sizes used Cohen’s d. Receiver operating characteristic analysis reported area under the curve, with values presented as max(AUC, 1 − AUC). Multiple-testing correction used the Benjamini–Hochberg false discovery rate method for feature-level comparisons. Point estimates are reported with 95% confidence intervals (CI) and *p*-values to two significant figures. Analyses were performed in Python 3.8 (scikit-learn, SciPy, statsmodels, pandas, imbalanced-learn). Analytical reproducibility (intra-run, complementary-DNA-synthesis, aliquot, inter-draw) and robustness to pre-analytical confounders (peri-sampling pharmacologic exposure and imaging) are reported in the Supplementary Materials. Decision curve analysis was performed in the matched per-protocol cohort to evaluate the net clinical benefit of Foretell Ovary and CA-125. Binary test classifications were fixed using the pre-specified clinical cutoffs (0.44 for Foretell Ovary and 35 U/mL for CA-125), and net benefit was calculated across threshold probabilities ranging from 1% to 50%.

### 2.10. Sample Size and Precision

No formal a priori power calculation was undertaken before the research. The sample size was determined by the number of eligible participants accrued at the six sites during the pre-specified enrollment window (September 2024 to March 2026), and the study was designed to estimate diagnostic accuracy with defined precision rather than to test a hypothesis. With 24 invasive ovarian cancers and 145 non-cancer participants in the independent per-protocol test set, the achieved two-sided 95% Clopper–Pearson interval half-widths were ±14.9 percentage points for sensitivity and ±5.4 percentage points for specificity. For reference, estimating a sensitivity of 87.5% to within ±15 percentage points requires 19 cases and to within ±10 percentage points requires 43 cases, whereas estimating a specificity of 89.0% to within ±5 percentage points requires 151 non-cancer participants. The test set therefore supports precise estimation of specificity and of the area under the curve (0.923; 95% CI 0.848–0.998, Hanley–McNeil method), more limited precision for sensitivity, and only exploratory precision for the stage-specific estimates, of which Stage I (*n* = 7) is the smallest. The matched head-to-head comparison with CA-125 (20 ovarian cancers) is likewise sized to detect only large paired differences: with an exact McNemar test at α = 0.05, 20 discordant pairs are required for 80% power when 80% of discordances favor one test, whereas the Stage I comparison yielded 4 discordant pairs. Comparative results are therefore reported as estimates with confidence intervals rather than as powered hypothesis tests, and a prospective study sized for a pre-specified difference in sensitivity is planned.

## 3. Results

### 3.1. Study Cohort

Of the 460 participants enrolled (training *n* = 279, independent test *n* = 181), 67 had ovarian cancer (65 with recorded FIGO Stage I–IV; two with stage not recorded) and 15 had borderline tumors, 197 benign gynecological conditions, and 181 asymptomatic controls (Table 1). Median age was 46.0 years (interquartile range 36.0–56.0); 99.3% were female. Among all 67 ovarian cancers, 17 (25.4%) were Stage I, 12 (17.9%) Stage II, 23 (34.3%) Stage III, and 13 (19.4%) Stage IV, with FIGO stage not recorded for the remaining two (3.0%). Histological subtype is described in Table S3.

### 3.2. Overall Diagnostic Performance

**Figure 1:**
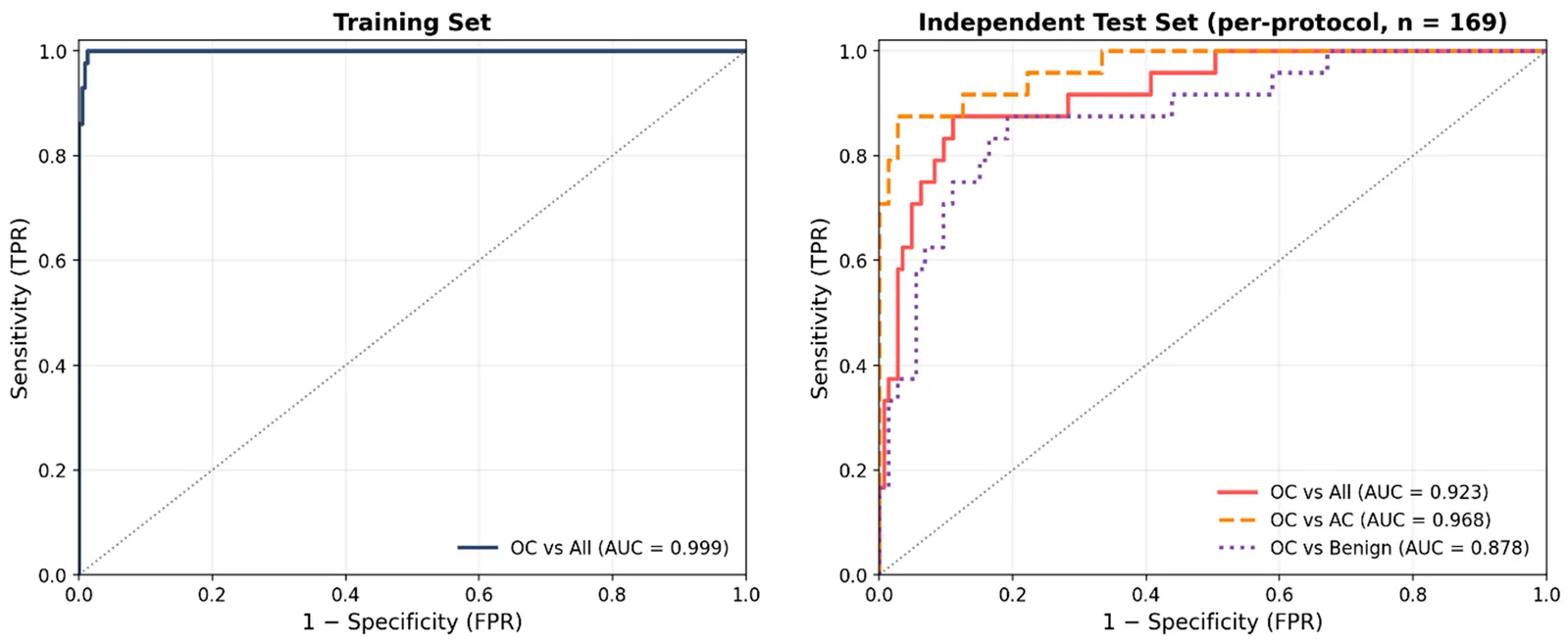
Receiver operating characteristic curves for Foretell Ovary in the training set (**left**) and the per-protocol independent test set (**right**). Test-set curves are shown for ovarian cancer versus all non-ovarian-cancer participants, versus asymptomatic controls only, and versus benign gynecological conditions only.

**Figure 2:**
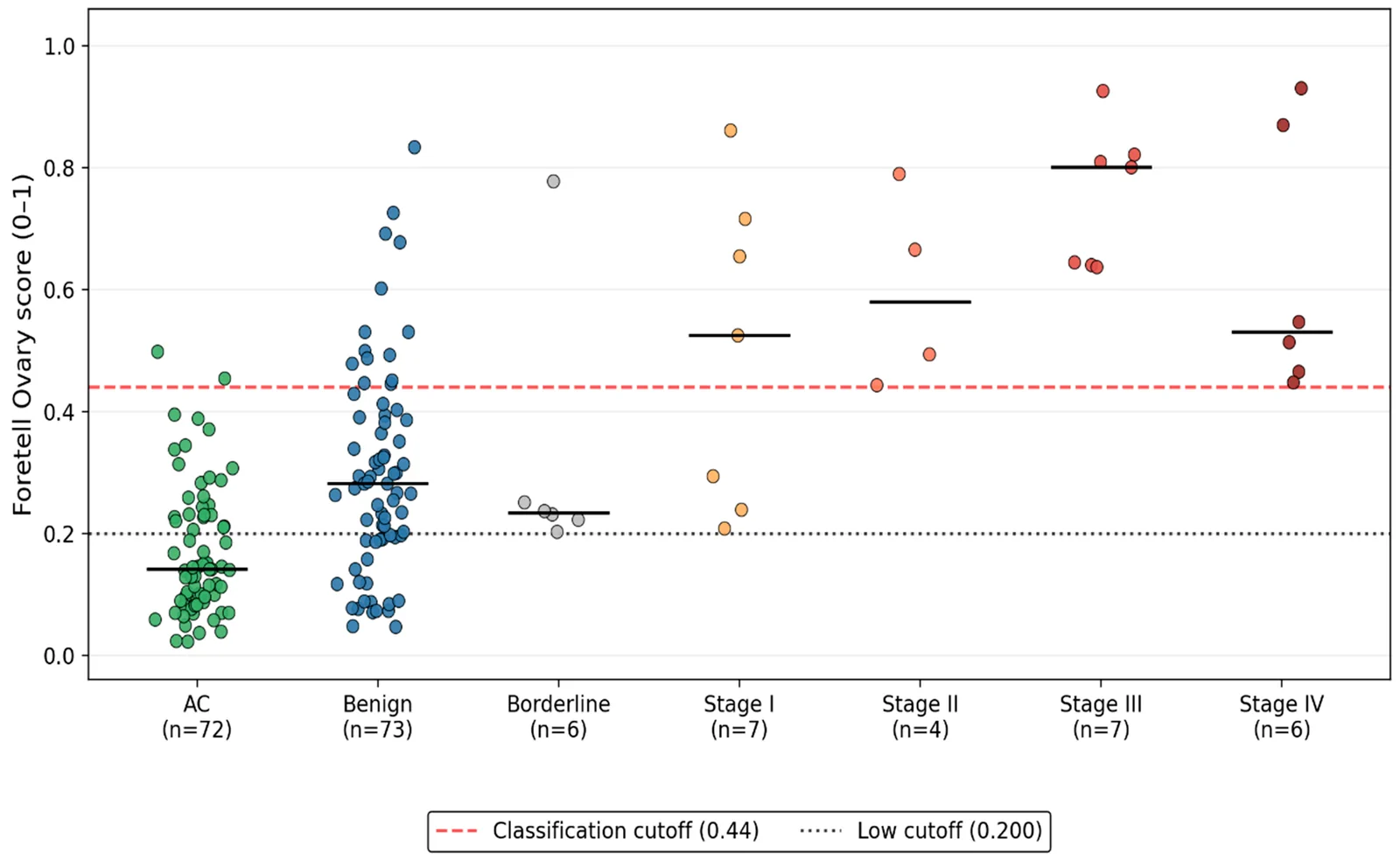
Model score distribution by ovarian cancer stage in the independent test set. The dashed red line indicates the classification cutoff (raw score 0.44); the dotted line indicates the low cutoff (0.200) separating low-risk from intermediate-risk grades. Borderline ovarian tumors are shown separately as a reference subgroup.

In the per-protocol test set (*n* = 169; ovarian cancer 24, non-ovarian-cancer 145), Foretell Ovary achieved an AUC of 0.923 overall, with subgroup AUC of 0.968 versus asymptomatic controls and 0.878 versus benign gynecological conditions (Figure 1). At the pre-frozen cutoff of 0.44, sensitivity was 87.5% (21/24; 95% CI 67.6–97.3%) and specificity 89.0% (129/145; 95% CI 82.7–93.6%), with positive predictive value of 56.8% and negative predictive value of 97.7%. Training- and independent-test performance with 95% bootstrap confidence intervals is given in Table S4, and the distribution of participants across the three risk grades in Table S5. Sensitivity for invasive Stage II–IV ovarian cancer was 100.0% (17/17); the three false-negative cases were all Stage I (Figure 2). Model calibration was assessed using a reliability curve and the Brier score (0.093 on a 0–1 scale, where 0 indicates perfect probabilistic accuracy); predicted probabilities modestly overestimated the observed ovarian-cancer frequency in the low-to-moderate probability range (~0.2–0.44), whereas predicted and observed frequencies were more closely aligned at higher predicted probabilities (≥0.5) (Supplementary Figure S1).

### 3.3. Stage-Stratified Performance

Score distributions by invasive ovarian cancer stage (Figure 2) showed Stage II and above concentrated above the 0.44 cutoff, while Stage I cases dispersed around it. The three false-negative Stage I cases were all retained at intermediate risk (range 0.208–0.295), preserving the safety-net principle of the three-tier scheme; no ovarian cancer case was assigned low risk in the test set (Supplementary Figure S2).

Stage-specific sensitivity in the independent test set, with 95% confidence intervals (Clopper–Pearson exact method), was 57.1% (4/7; 95% CI 18.4–90.1%) for Stage I, 100.0% (4/4; 95% CI 39.8–100.0%) for Stage II, 100.0% (7/7; 95% CI 59.0–100.0%) for Stage III, and 100.0% (6/6; 95% CI 54.1–100.0%) for Stage IV, with a combined Stage II–IV sensitivity of 100.0% (17/17; 95% CI 80.5–100.0%) (Table S6). The wide confidence interval for the Stage I estimate reflects the small subgroup size (*n* = 7) and indicates that this point estimate should be interpreted with caution pending validation in a larger cohort. The individual false-negative cases are listed in Table S7.

Sensitivity did not differ appreciably between the two histological subtypes with adequate test-set representation (high-grade serous carcinoma 8/8, 100.0%; unspecified epithelial subtype 9/10, 90.0%); all remaining subtypes were represented by one or two test-set cases each (Table S3).

### 3.4. Head-to-Head Comparison with CA-125

**Figure 3:**
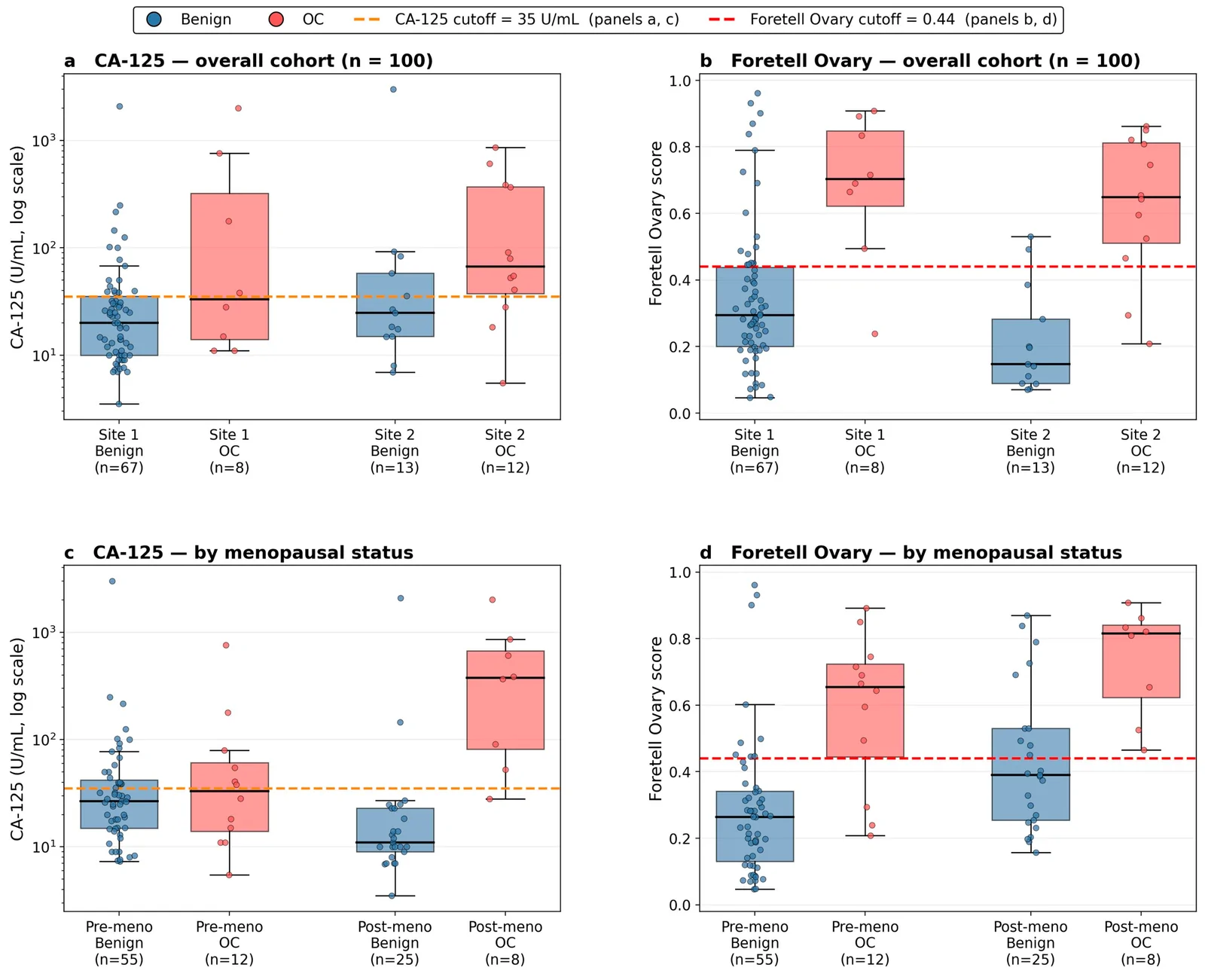
Score distributions of CA-125 and Foretell Ovary in the matched per-protocol cohort. (**a**) CA-125 distribution overall (Site 1/Site 2, benign vs. ovarian cancer). (**b**) Foretell Ovary score overall, same stratification. (**c**) CA-125 distribution stratified by menopausal status. (**d**) Foretell Ovary score stratified by menopausal status. CA-125 dashed line: 35 U/mL clinical cutoff; Foretell Ovary dashed line: 0.44 classification cutoff. Overall *n* = 100 (ovarian cancer 20, benign 80); pre-menopausal *n* = 67; post-menopausal *n* = 33.

Foretell Ovary was compared head-to-head against CA-125 (clinical cutoff 35 U/mL) in 105 participants with concurrent measurements (ovarian cancer 20, benign 85). After per-protocol exclusion of five benign quality-control failures, the primary comparative cohort comprised 100 participants (ovarian cancer 20, benign 80); the intent-to-test reference (*n* = 105) is reported in parallel. Foretell Ovary outperformed CA-125 across all metrics: sensitivity 85.0% (17/20) versus 65.0% (13/20) (+20.0 percentage points), specificity 76.2% versus 72.5% (+3.7 percentage points), and AUC 0.839 versus 0.723 (+0.116). Positive predictive value rose from 37.1% to 47.2% and negative predictive value from 89.2% to 95.3%. The separation between ovarian cancer and benign samples was visibly larger on the Foretell Ovary axis than on the CA-125 axis (Figure 3). The full metric-by-metric comparison is given in Table S8.

A stage-specific head-to-head comparison was performed in the largest stage-specific subgroup of invasive ovarian cancer within the matched cohort, Stage I (*n* = 10; 7 from the independent test set and three from the training set). Foretell Ovary and CA-125 showed identical sensitivity in Stage I (70.0%, 7/10; 95% CI 34.8–93.3% for both tests), although the two assays were discordant in four of the 10 cases, with each uniquely detecting two cases missed by the other (McNemar exact *p* = 1.00). The higher overall sensitivity of Foretell Ovary relative to CA-125 in this cohort (85.0% vs. 65.0%) was observed mainly in Stage II ovarian cancer (100.0%, 5/5, vs. 40.0%, 2/5), whereas Stage I sensitivity was identical between the two tests. Stage III and IV subgroups (*n* = 2 each) were too small to permit reliable stage-specific comparisons. Full stage-stratified counts and corresponding 95% confidence intervals are reported in Supplementary Table S2.

### 3.5. Menopausal Subgroup and Combination Strategies

**Figure 4:**
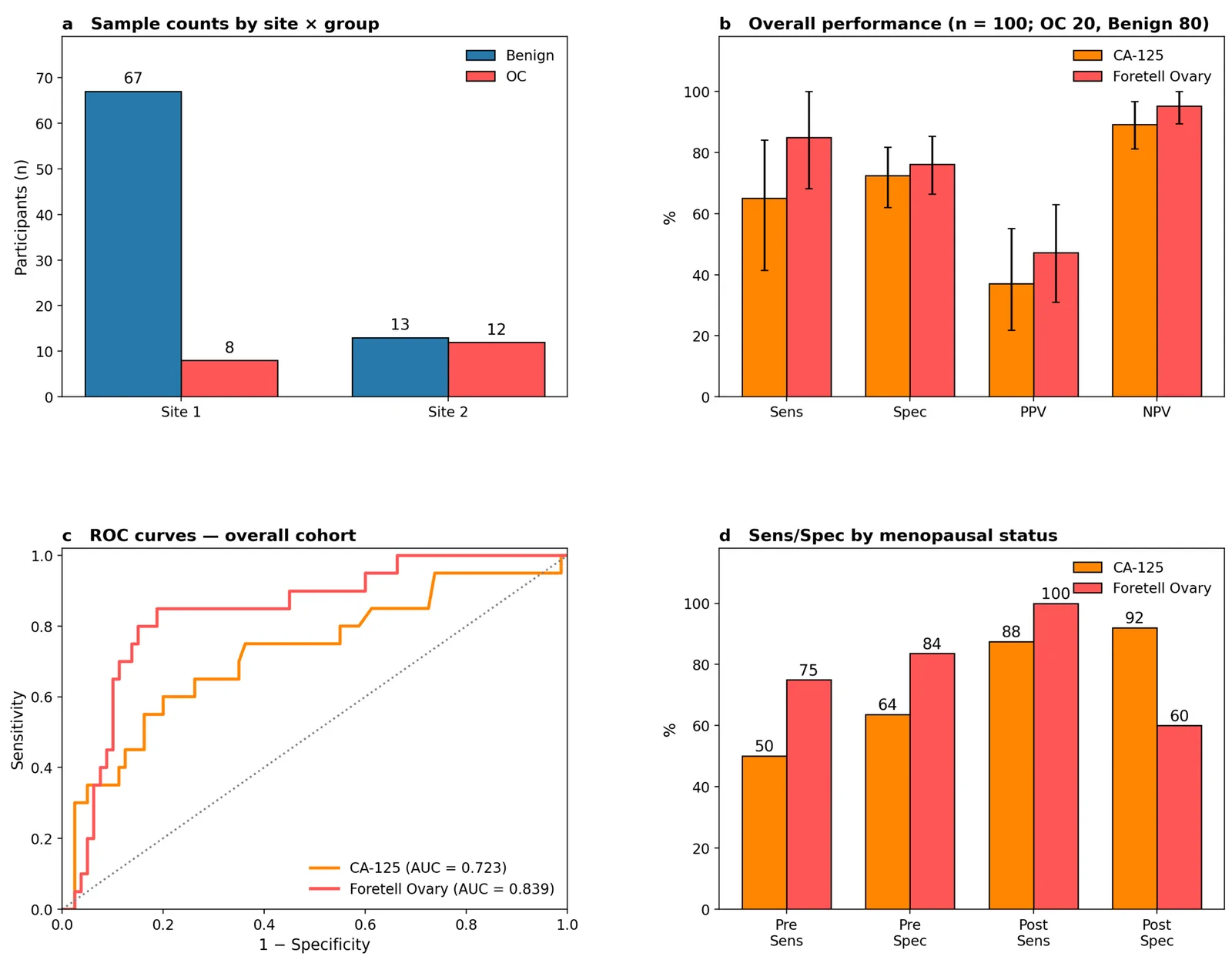
Diagnostic-performance summary of CA-125 versus Foretell Ovary. (**a**) Sample counts by site and group in the matched cohort. (**b**) Overall sensitivity, specificity, positive predictive value, and negative predictive value (*n* = 100; ovarian cancer 20, benign 80) with 95% bootstrap confidence intervals. (**c**) Receiver operating characteristic curves on the overall cohort. (**d**) Sensitivity and specificity stratified by menopausal status. CA-125 cutoff = 35 U/mL; Foretell Ovary cutoff = 0.44.

Subgroup analyses by menopausal status revealed differential patterns. In pre-menopausal participants (ovarian cancer 12, benign 55), Foretell Ovary showed substantially higher sensitivity than CA-125 (75.0% vs. 50.0%, +25.0 percentage points) and specificity (83.6% vs. 63.6%, +20.0 percentage points). In post-menopausal participants (ovarian cancer 8, benign 25), Foretell Ovary achieved 100.0% sensitivity versus 87.5% for CA-125, while CA-125 retained higher specificity (92.0% vs. 60.0%); subgroup metrics are summarized in Table S9. The overall performance summary, including 95% confidence intervals and receiver operating characteristic curves, is shown in Figure 4. Logical combination offered flexible operating points: an OR rule reached 95.0% sensitivity (19/20) at 51.2% specificity, while an AND rule reached 97.5% specificity at 55.0% sensitivity (Table S10). The two tests captured largely orthogonal information (Spearman ρ = 0.045, *p* = 0.65; Supplementary Figure S3).

### 3.6. Feature-Level and Robustness Analyses

At the feature level, combination features exceeded any single marker in discriminative capacity: LGALS3BP-a × hematocrit/mean corpuscular hemoglobin concentration and LGALS3BP-a × hematocrit/neutrophil percentage reached AUC 0.89, versus a best single-marker AUC of 0.78 (FCGR2A-b). Single-marker and combination-feature details are in Supplementary Tables S11 and S12 and Figure S4, and the complete blood count profile by diagnostic group is in Table S13. Sequential exclusion of pre-analytical confounder samples (peri-sampling pharmacologic exposure or imaging) changed the test-set AUC by less than 0.013 (Supplementary Section S3, Tables S14 and S15), and analytical reproducibility was 100.0% binary concordance across all four tiers (intra-run, complementary-DNA-synthesis, aliquot, and inter-draw; Supplementary Section S4, Tables S16–S19).

### 3.7. Decision Curve Analysis

**Figure 5:**
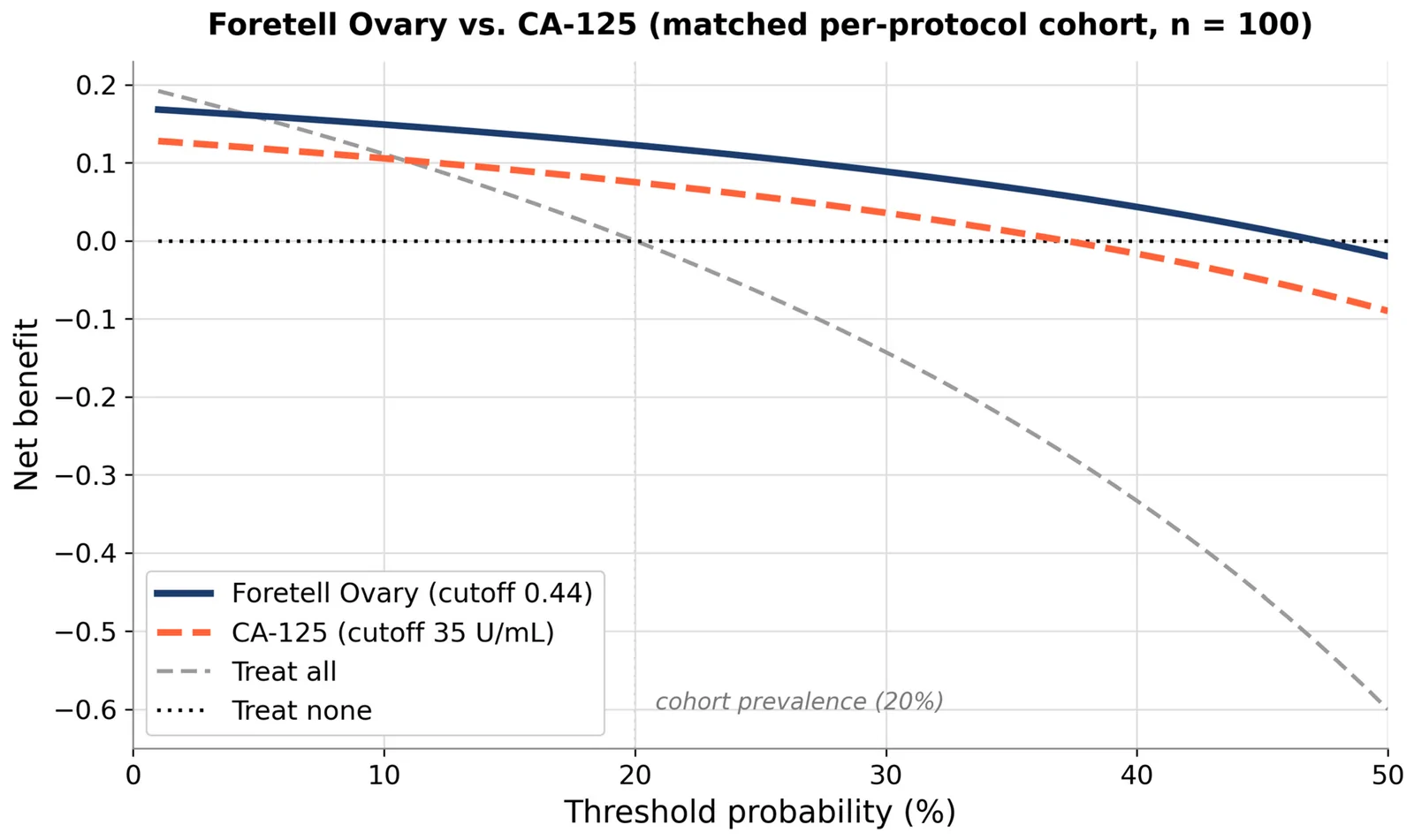
Decision curve analysis comparing Foretell Ovary and CA-125 in the matched per-protocol cohort (*n* = 100; ovarian cancer 20, benign 80). Net benefit is plotted against threshold probability for Foretell Ovary (cutoff 0.44), CA-125 (cutoff 35 U/mL), a treat-all strategy, and a treat-none reference line. The vertical dotted line marks the ovarian-cancer prevalence within this matched subset (20%).

Decision curve analysis was performed in the matched per-protocol cohort (*n* = 100; ovarian cancer 20, benign 80) to compare the net clinical benefit of Foretell Ovary and CA-125 at their pre-specified clinical cutoffs, rather than as continuously re-thresholded risk scores. Across the full range of threshold probabilities tested (1–50%), Foretell Ovary’s net benefit exceeded that of CA-125 at every threshold, and remained positive (superior to a treat-none strategy) up to a threshold probability of 47%, compared with 37% for CA-125. At the matched cohort’s own ovarian-cancer prevalence (20%), Foretell Ovary showed a higher net benefit than both CA-125 (0.123 vs. 0.075) and a treat-all strategy, which crossed zero net benefit at exactly this threshold (Figure 5).

## 4. Discussion

In this multi-site prospective cohort of 460 South Korean participants, Foretell Ovary—an ensemble classifier integrating eight platelet RNA markers with routine complete blood count parameters and 30 marker-by-hematology combination features—achieved AUC of 0.923, sensitivity of 87.5%, and specificity of 89.0% in the per-protocol independent test set (*n* = 169), with 100.0% sensitivity for invasive Stage II–IV disease and 97.7% negative predictive value. In a head-to-head matched subset (*n* = 100), Foretell Ovary outperformed CA-125 on every evaluated metric, with the largest gains in pre-menopausal participants (+25 percentage points in sensitivity, +20 percentage points in specificity). The three-tier risk-grading scheme preserved an explicit safety net: no ovarian cancer case in the test set was assigned to the low-risk grade, and the three Stage I false-negative cases below the 0.44 binary cutoff were all retained at intermediate risk above the 0.200 low-risk threshold.

Our per-protocol AUC (0.923) is consistent with the tumor-educated-platelet ovarian cancer literature: Gao et al. (2023) reported AUC 0.918 (*n* = 761, improving to 0.922 with CA-125) [13], Best et al. (2015) achieved 96% accuracy across six cancer types [11], and In ’t Veld et al. (2022) reached 99% specificity across 18 cancer types in 2351 individuals [12]. The present study uses a focused panel of eight RT-qPCR assays (rather than transcriptome-wide RNA sequencing) integrated with the routine complete blood count, providing a practical advantage for potential implementation in clinical laboratories. The +25 percentage-point pre-menopausal sensitivity gain over CA-125 may help address the well-known limitation of CA-125 in this subgroup, and contrasts with the Risk of Ovarian Malignancy Algorithm [6], which targets benign-versus-malignant discrimination in patients with an existing pelvic mass rather than screening.

The eight markers span six genes that report distinct biological axes of platelet–tumor communication. IGFBP2 reports IGF/PI3K-AKT growth-axis activity; serum IGFBP2 has shown diagnostic AUC 0.815 (0.904 in advanced disease) [19] and IGFBP2-containing panels detect Type I and Type II cancers missed by CA-125 with a 5–6-month lead time [20]. LGALS3BP (also termed 90K/Mac-2BP) is positive in 78% of ovarian cancer cases versus 49% for CA-125 alone (86% sensitivity in combination) [21], and FCGR2A polymorphisms predict response to anti-folate-receptor-α therapy [22]. IL1R2 is a decoy receptor for IL-1 cytokines whose blockade synergizes with anti-PD-1 therapy [23]; in our cohort, IL1R2 expression was lowest in asymptomatic controls and elevated in both benign and ovarian cancer samples, consistent with reporting of ovarian-disease state rather than malignancy alone. Two assays target transcribed pseudogenes consistent with the tumor-educated-platelet framework: WHAMMP3-a (a WHAMM pseudogene at 15q11.2) and LOC100420587-a (an SHCBP1 pseudogene at the 19q12/CCNE1 amplicon, amplified in ~36% of high-grade serous ovarian cancers [24]) were both down-regulated in ovarian cancer relative to benign disease; SHCBP1 itself drives AKT/mTOR-dependent cisplatin resistance [25]. The LOC100420587-a assay detects a non-canonical exon-3-to-exon-5 transcript (exon-4 skipping), a candidate substrate for signal-dependent pre-mRNA splicing that anucleate platelets perform [7]; pseudogene transcripts are well documented in the platelet transcriptome [26]. The down-regulation of these pseudogenes combined with up-regulation of IGFBP2, LGALS3BP, and FCGR2A is consistent with the model reading a coordinated tumor-educated-platelet reprogramming signature.

The final panel of eight markers was not a pre-specified target but the outcome of the staged assay qualification workflow (Section 2.4). Each qualification stage progressively eliminated candidates that failed predefined technical stability criteria, and no additional candidates satisfied all qualification requirements beyond the eight that passed the final reference-standard-based stability testing. One marker, WHAMMP3-a, did not reach univariate significance in the validation cohort (*p* = 0.359 vs. asymptomatic controls; *p* = 0.496 vs. benign gynecological conditions; Supplementary Table S11) but was retained because it successfully passed the complete assay qualification workflow and contributed to the combination-feature sub-model (Section 2.6), where marker selection was based on multivariable predictive performance rather than individual univariate discriminative power.

Strengths of this study include multi-site enrollment across six sites, a pre-specified ACTB cycle threshold ≤ 28 per-protocol rule with parallel intent-to-test reporting, a single pre-specified train/test partition with no model re-tuning on the test set, head-to-head matched comparison with CA-125, and use of routine complete blood count rather than research-grade instrumentation—supporting future clinical implementation. Reproducibility was 100.0% binary concordance across all four tiers and AUC varied by less than 0.013 across all confounder-exclusion scenarios. Limitations include enrollment from a single country (South Korea), which may limit the generalizability of these findings to other ethnic, geographic, and healthcare settings. Because the head-to-head comparison was restricted to participants with available paired CA-125 and Foretell Ovary measurements, the comparison subset may not fully represent the overall study population. External validation in diverse populations and clinical settings is therefore needed to evaluate the transportability of Foretell Ovary across different practice environments. The cohort is enriched for tertiary-care presentations, and the reported positive predictive value should be interpreted at the ovarian-cancer prevalence of the per-protocol test set (24/169, 14.2%). The positive predictive value of 56.8% is a genuine limitation of the test in its present form: at the deployed operating point, 16 of the 37 positive results (43.2%) were false positives, and the value would be lower still in a screening population of lower prevalence. The CA-125 matched subset (*n* = 100) is modest, with small post-menopausal numbers (*n* = 33); broader external validation of menopausal-subgroup performance is needed.

The +25 percentage-point pre-menopausal sensitivity gain over CA-125 may help address a well-known limitation of CA-125 in this subgroup and suggests that Foretell Ovary may serve as a non-invasive complement to, rather than a replacement for, CA-125. Because Foretell Ovary and CA-125 capture largely orthogonal information, their outputs can be combined and the operating point tailored to the clinical objective—Foretell Ovary alone, CA-125 alone, OR combination for case detection, or AND combination for confirmatory specificity. Combining the two can also improve the moderate positive predictive value with Foretell Ovary alone (Supplementary Table S10). Because no blood-based test with adequate standalone performance is currently established for ovarian cancer screening, a test used alongside CA-125 rather than in place of it may still offer clinical value at this stage, and raising the positive predictive value is an explicit objective of our follow-up work—through expansion of the marker panel, recalibration in larger and lower-prevalence cohorts, and prospective evaluation of the combination rules reported here. The three-tier risk-grading scheme provides a graded risk output that is conceptually aligned with how screening tests are used in practice. Prospective interventional studies will be important to determine how this approach performs in real-world clinical settings and to further evaluate its clinical utility.

## 5. Conclusions

Foretell Ovary, a blood-based ensemble classifier that integrates eight platelet RNA markers with routine complete blood count parameters and marker-by-hematology combination features, achieved an AUC of 0.923 with sensitivity of 87.5% and specificity of 89.0% in an independent multi-site test set, and outperformed CA-125 in a head-to-head matched comparison with the largest gains in pre-menopausal participants. These findings support Foretell Ovary as a feasible non-invasive screening complement to CA-125, particularly in the pre-menopausal population where CA-125 has historically underperformed.

## Figures and Tables

**Table 1:** Baseline characteristics of study participants.

Characteristic	Overall (*n* = 460)	Ovarian Cancer (*n* = 67)	Borderline (*n* = 15)	Benign (*n* = 197)	Asymptomatic (*n* = 181)	*p*-Value
**Training set**	279 (60.7%)	43 (64.2%)	9 (60.0%)	118 (59.9%)	109 (60.2%)	—
**Test set**	181 (39.3%)	24 (35.8%)	6 (40.0%)	79 (40.1%)	72 (39.8%)	—
**Age, years**	46.0 (36.0–56.0)	56.0 (49.0–66.0)	46.0 (34.0–59.0)	45.0 (36.0–53.0)	42.0 (30.0–54.0)	<0.001
**Sex, female**	457 (99.3%)	67 (100%)	15 (100%)	197 (100%)	178 (98.3%)	—
**Site 1**	222 (48.3%)	42 (18.9%)	10 (4.5%)	152 (68.5%)	18 (8.1%)	—
**Site 2**	129 (28.0%)	3 (2.3%)	—	2 (1.6%)	124 (96.1%)	—
**Site 3**	54 (11.7%)	15 (27.8%)	5 (9.3%)	34 (63.0%)	—	—
**Site 4**	28 (6.1%)	7 (25.0%)	—	9 (32.1%)	12 (42.9%)	—
**Site 5**	15 (3.3%)	—	—	—	15 (100%)	—
**Site 6**	12 (2.6%)	—	—	—	12 (100%)	—
**FIGO Stage I**	—	17 (25.4%)	—	—	—	—
**FIGO Stage II**	—	12 (17.9%)	—	—	—	—
**FIGO Stage III**	—	23 (34.3%)	—	—	—	—
**FIGO Stage IV**	—	13 (19.4%)	—	—	—	—
**FIGO Stage not recorded**	—	2 (3.0%)	—	—	—	—

Data presented as median (interquartile range) for continuous variables and *n* (%) for categorical variables. *p*-values from Kruskal–Wallis test across the ovarian cancer, benign, and asymptomatic-control groups (borderline tumors excluded from statistical comparison). Site 2 is the asymptomatic-control enrollment cohort under a single Handong Global University IRB approval. Site-row percentages are of each site’s total; all other percentages are of each column’s group total. FIGO Stage percentages are of all 67 ovarian cancer cases, including the two cases (both in the training set) with no FIGO stage recorded, shown as a separate row. Borderline ovarian tumors (*n* = 15) are reported as a separate reference subgroup, not pooled with invasive ovarian cancer and not included in primary performance analyses.

## Data Availability

The data presented in this study are available on reasonable request from the corresponding author. The data are not publicly available due to privacy and ethical restrictions imposed by the participating institutions and the data-sharing agreement with Foretell My Health, Inc.

## Supplementary Materials

The following supporting information can be downloaded at https://www.mdpi.com/article/10.3390/cancers18162629/s1, Table S1: Individual sub-model versus ensemble performance; Table S2: Stage-stratified diagnostic performance: Foretell Ovary versus CA-125 in the matched per-protocol cohort; Table S3: Histological subtype distribution and subtype-stratified test-set performance for invasive ovarian cancer; Table S4: Diagnostic performance of Foretell Ovary in the training set and independent test set; Table S5: Three-tier risk grade distribution across diagnostic groups in the independent test set; Table S6: Stage-stratified sensitivity in the independent test set; Table S7: False-negative cases in the independent test set; Table S8: Overall diagnostic performance: Foretell Ovary versus CA-125; Table S9: Diagnostic performance by menopausal status; Table S10: Combination strategy performance; Table S11: Individual platelet RNA marker discrimination performance in the independent test set; Table S12: Top 10 combination features of the combination-feature logistic-regression sub-model; Table S13: Complete blood count parameters and derived hematological indices across participant groups; Table S14: Model performance across screening-failure-exclusion scenarios; Table S15: Overlap between misclassified cases and screening-confounder samples; Table S16: Intra-run PCR repeatability (same complementary DNA, same plate); Table S17: Complementary-DNA-synthesis repeatability (5 independent reverse transcriptions per subject from the same RNA); Table S18: Aliquot reproducibility (independent platelet-RNA aliquots from the same blood draw); Table S19: Inter-draw reproducibility in healthy volunteers (1–6 month intervals); Figure S1: Calibration plot comparing; Figure S2a: Three-tier risk grade distribution across diagnostic groups; Figure S2b: Test-set sample distribution by Foretell Ovary score; Figure S3: Scatter plot of CA-125; Figure S4a: Single-marker AUC summary for ovarian cancer versus asymptomatic control and Figure S4b: Single-marker AUC summary for ovarian cancer versus benign gynecological conditions. Test set.

## Abbreviations

The following abbreviations are used in this manuscript:

AUC	Area under the receiver operating characteristic curve
CA-125	Cancer antigen 125
CBC	Complete blood count
CI	Confidence interval
Cq	Cycle threshold (quantification cycle)
FIGO	International Federation of Gynecology and Obstetrics
IRB	Institutional Review Board
NPV	Negative predictive value
PPV	Positive predictive value
RT-qPCR	Reverse-transcription quantitative polymerase chain reaction
SMOTE	Synthetic minority over-sampling technique
