# Supplementary material for: Foretell Ovary: A Blood-Based Ensemble Algorithm Integrating Tumor-Educated Platelet RNA and Routine Hematology for Non-Invasive Ovarian Cancer Screening
Source: Cancers (Basel). 2026 Aug 14;18(16):2629. doi: 10.3390/cancers18162629 (PMC13511248; doi:10.3390/cancers18162629)
Supplement: Supplementary file 1 [file cancers-18-02629-s001.zip › cancers-4454135-supplementary.pdf]

Supplementary Information

Foretell Ovary: a blood-based ensemble algorithm for non-invasive ovarian cancer screening

Contents

S1. Stage-, subtype-, and sub-model-stratified analyses

Tables S1–S3 (sub-model versus ensemble; stage-stratified CA-125 comparison; histological subtype)

S2. Expanded performance and feature analyses

Tables S4–S13 (overall performance; three-tier risk grading; stage-stratified sensitivity; false-negative cases; CA-125 comparison; combination strategies; single-marker discrimination; combination features; CBC parameters)

Figures S1–S4 (calibration; risk-grade distribution; CA-125 vs Foretell Ovary scatter; single-marker AUC)

S3. Robustness to pre-analytical confounders

Tables S14–S15

S4. Analytical reproducibility and repeatability

Tables S16–S19

S1. Stage-, subtype-, and sub-model-stratified analyses

Table S1. Individual sub-model versus ensemble diagnostic performance (per-protocol independent test set, n = 169; ovarian cancer 24), evaluated at the deployed classification cutoff (0.44).

Sub-model	Ensemble weight	AUC	Sensitivity	Specificity
Random forest (raw features) alone	0.60	0.926	66.7% (16/24)	92.4% (134/145)
Logistic regression (raw features) alone	0.28	0.908	87.5% (21/24)	82.1% (119/145)
Logistic regression (combination features) alone	0.12	0.782	62.5% (15/24)	77.2% (112/145)
**Ensemble (deployed model)**	—	**0.923**	**87.5% (21/24)**	**89.0% (129/145)**

Sensitivity, specificity, and AUC for each sub-model were computed independently at the same fixed classification cutoff (0.44) used for the deployed ensemble, to isolate the effect of combining sub-models from any difference in operating point. Balanced accuracy (mean of sensitivity and specificity) was 79.6%, 84.8%, 69.9%, and 88.2% for the random forest, raw-feature logistic regression, combination-feature logistic regression, and ensemble, respectively.

Table S2. Stage-stratified diagnostic performance: Foretell Ovary versus CA-125 in the matched per-protocol cohort (invasive ovarian cancer with recorded FIGO stage, n = 19 of 20; one additional case with stage not recorded is included in the overall n = 20 figures reported in §3.4 but omitted here). Sensitivities are reported with 95% confidence intervals (Clopper-Pearson exact method). Added in response to Reviewer 1 (R1-4).

Stage	N	CA-125 Detected/N, % (95% CI)	Foretell Ovary Detected/N, % (95% CI)
Stage I	10	7/10, 70.0% [34.8–93.3%]	7/10, 70.0% [34.8–93.3%]
Stage II	5	2/5, 40.0% [5.3–85.3%]	5/5, 100.0% [47.8–100.0%]
Stage III	2	2/2, 100.0% [15.8–100.0%]	2/2, 100.0% [15.8–100.0%]
Stage IV	2	2/2, 100.0% [15.8–100.0%]	2/2, 100.0% [15.8–100.0%]

Note: the Stage I row (n = 10) comprises 7 independent test-set cases and 3 training-set cases with concurrent CA-125 measurements, consistent with the matched-cohort composition described in §2.8; all comparisons use the frozen Foretell Ovary model score with no re-fitting.

Table S3. Histological subtype distribution and subtype-stratified test-set performance for invasive ovarian cancer (per-protocol independent test set, n = 24; full cohort, n = 65 of the 67 ovarian cancers, the 2 cases without recorded FIGO stage being excluded; plus 15 borderline tumors as a reference subgroup).

Histological subtype	Overall n (%)	Test-set n	Detected	Sensitivity
Serous, high-grade (HGSC)	25 (38.5%)	8	8	100.0%
Epithelial OC, not otherwise specified	19 (29.2%)	10	9	90.0%
Histology not obtained/unknown	6 (9.2%)	2	1	50.0%
Endometrioid	4 (6.2%)	0	—	—
Clear cell	3 (4.6%)	0	—	—
Non-epithelial (neuroendocrine/sex cord-stromal)	2 (3.1%)	0	—	—
Carcinosarcoma	2 (3.1%)	0	—	—
Other definitive subtype (Brenner tumor/primary peritoneal)	1 (1.5%)	1	0	0.0%
Serous, not otherwise specified	1 (1.5%)	1	1	100.0%
Mucinous	1 (1.5%)	1	1	100.0%
Serous, low-grade (LGSC)	1 (1.5%)	1	1	100.0%
**Total invasive**	**65 (100.0%)**	**24**	**21**	**87.5%**
Borderline tumors (reference only)—Serous NOS 6, Epithelial unspecified 5, Mucinous 3, Endometrioid 1	15 (100.0%)	—	—	—

Histological subtype was assigned from centrally reviewed pathology records; borderline tumors are reported separately as a reference subgroup and are not included in the invasive-subtype denominators or sensitivity calculations.

S2. Expanded performance and feature analyses

This section provides the detailed tables and figures supporting the main-manuscript results, including the single-marker univariate performance, the top combination features of the logistic-regression sub-model on combination features, the full complete-blood-count parameter panel, the overall training/test-set diagnostic performance, the three-tier risk grading distribution, stage-stratified sensitivity, false-negative-case details, and the CA-125 comparison (overall, by menopausal status, and combination strategies).

Table S4. Diagnostic performance of Foretell Ovary in the training set and independent test set at the pre-frozen classification cutoff of 0.44, with 95% bootstrap confidence intervals.

Metric	Training set (N = 270, OC = 43)	Test set (N = 169, OC = 24)
AUC	0.999	0.923
OC vs AC	—	0.968
OC vs Benign	—	0.878
Sensitivity	100.0% (43/43)	87.5% (21/24)
Specificity (overall)	96.9% (220/227)	89.0% (129/145)
Specificity vs Benign	94.1% (111/118)	80.8% (59/73)
Specificity vs AC	100.0% (109/109)	97.2% (70/72)
Balanced accuracy	0.985	0.882
PPV	86.0%	56.8%
NPV	100.0%	97.7%

Table S5. Three-tier risk grade distribution across diagnostic groups in the independent test set. Grades are defined by raw model output: low-risk (raw < 0.200), intermediate-risk (0.200 ≤ raw < 0.44), high-risk (raw ≥ 0.44).

Diagnostic group	Low-risk (score < 0.200)	Intermediate-risk (0.200 ≤ score < 0.44)	High-risk (score ≥ 0.44)	Total
OC (invasive)	0	3 (12.5%)	21 (87.5%)	24
Benign	23 (31.5%)	36 (49.3%)	14 (19.2%)	73
Asymptomatic control (AC)	47 (65.3%)	23 (31.9%)	2 (2.8%)	72
Borderline ovarian tumor*	0	5 (83.3%)	1 (16.7%)	6

Table S6. Stage-stratified sensitivity in the independent test set (invasive ovarian cancer staged per FIGO 2014), with 95% confidence intervals (Clopper-Pearson exact method).

Stage	N	Detected	Sensitivity	95% CI
Stage I	7	4	57.1%	18.4–90.1%
Stage II	4	4	100.0%	39.8–100.0%
Stage III	7	7	100.0%	59.0–100.0%
Stage IV	6	6	100.0%	54.1–100.0%
**Stage II-IV (total)**	**17**	**17**	**100.0%**	80.5–100.0%
Borderline (reference only)	6	1	16.7%	0.4–64.1%

Table S7. False-negative cases in the independent test set. Sample identifiers are anonymized in keeping with the manuscript convention.

Sample	Stage	Score (0-1)	Distance to cutoff
FN-Case-01	I	0.295	-0.145
FN-Case-02	I	0.239	-0.201
FN-Case-03	I	0.208	-0.232

Table S8. Overall diagnostic performance: Foretell Ovary versus CA-125 (matched cohort, per-protocol n = 100; ovarian cancer 20, benign 80).

Metric	CA-125	Foretell Ovary	Difference
Sensitivity	65.0%	85.0%	+20.0%p
Specificity	72.5%	76.2%	+3.7%p
PPV	37.1%	47.2%	+10.1%p
NPV	89.2%	95.3%	+6.1%p
AUC	0.723	0.839	+0.116

Table S9. Diagnostic performance by menopausal status: Foretell Ovary versus CA-125.

Subgroup	Metric	CA-125	Foretell Ovary	Difference
Pre-menopausal(OC=12, Ben=55)	Sensitivity	50.0%	75.0%	+25.0%p
	Specificity	63.6%	83.6%	+20.0%p
Post-menopausal(OC=8, Ben=25)	Sensitivity	87.5%	100.0%	+12.5%p
	Specificity	92.0%	60.0%	-32.0%p

Table S10. Combination strategy performance: Foretell Ovary and CA-125 (matched per-protocol cohort, n = 100; intent-to-test reference n = 105). OR rule: positive if either test is positive. AND rule: positive only if both tests are positive.

Strategy	Rule	Sensitivity	Specificity	PPV	NPV
CA-125 alone	Positive if CA-125 > 35 U/mL	65.0%	72.5%	37.1%	89.2%
Foretell Ovary alone	Positive if score > 0.44	85.0%	76.2%	47.2%	95.3%
**OR combination**	**Positive if either test positive**	**95.0%**	**51.2%**	**32.8%**	**97.6%**
**AND combination**	**Positive only if both tests positive**	**55.0%**	**97.5%**	**84.6%**	**89.7%**

Table S11. Individual platelet RNA marker discrimination performance in the independent test set (per-protocol, n = 169; ovarian cancer 24, benign 73, asymptomatic control 72). AUC values reported separately against asymptomatic controls, benign gynecological conditions, and combined non-ovarian-cancer comparators, with Cohen's d effect sizes and p values from Mann–Whitney U tests.

Marker	AUC (vs AC)	AUC (vs Benign)	AUC (vs non-OC)	p-value (vs AC)	p-value (vs Benign)	Cohen's d (vs AC)	Cohen's d (vs Benign)
IGFBP2-a	0.587	0.557	0.572	0.203	0.410	-0.11	+0.03
IGFBP2-b	0.615	0.533	0.573	0.095	0.637	-0.31	-0.07
IL1R2-a	0.722	0.458	0.589	0.001	0.539	-0.67	+0.36
IL1R2-b	0.689	0.477	0.605	0.006	0.741	-0.47	+0.12
LGALS3BP-a	0.748	0.727	0.737	<0.001	<0.001	-0.74	-0.72
WHAMMP3-a	0.563	0.547	0.508	0.359	0.496	+0.22	+0.13
LOC100420587-a	0.519	0.757	0.639	0.790	<0.001	+0.21	+1.21
FCGR2A-b	0.690	0.390	0.351	0.006	0.109	+0.11	+0.09

Table S12. Top 10 combination features of the combination-feature logistic-regression sub-model, ranked by absolute coefficient magnitude, with single-feature discrimination performance.

Combination feature	LR_combo coef (signed)	AUC (OC vs non-OC)	AUC (OC vs AC)	AUC (OC vs Benign)	Cohen's d (OC vs non-OC)
HGB − HCT	-1.155	0.868	0.937	0.800	+1.70
HGB − MCHC	-1.023	0.867	0.905	0.829	-1.73
IGFBP2-a (ΔCq) × LYM#	+0.906	0.788	0.872	0.706	-0.96
IGFBP2-b (ΔCq) + HCT	-0.794	0.833	0.931	0.737	-1.37
LGALS3BP-a (ΔCq) × HCT / NEUT%	+0.746	0.888	0.950	0.823	-1.28
IGFBP2-a (ΔCq) × HCT / NEUT%	-0.621	0.773	0.846	0.695	-0.86
LGALS3BP-a (ΔCq) × HCT / MCHC	-0.615	0.893	0.936	0.851	-1.32
IGFBP2-a (ΔCq) × IGFBP2-b (ΔCq) / PLR	-0.524	0.742	0.821	0.663	-0.63
IGFBP2-a (ΔCq) + HCT	-0.483	0.805	0.893	0.718	-1.33
IGFBP2-a (ΔCq) + IGFBP2-b (ΔCq) × LYM#	-0.469	0.800	0.891	0.711	-0.97

Table S13. Complete blood count parameters and derived hematological indices across participant groups (ovarian cancer pooled with borderline tumors, benign gynecological conditions, asymptomatic control). Data are presented as median (interquartile range); p values from Kruskal–Wallis test across three groups.

CBC parameter / derived index	Overall (N=460)	OC and Borderline (n=82)	Benign (n=197)	AC (n=181)	p-value
WBC (x10^3/uL)	5.7 (4.6-7.0)	5.8 (4.3-7.1)	5.3 (4.1-6.9)	6.1 (4.9-6.9)	0.016
RBC (x10^6/uL)	4.2 (3.9-4.5)	3.9 (3.5-4.2)	4.0 (3.8-4.3)	4.4 (4.2-4.6)	<0.001
Hemoglobin (g/dL)	12.4 (11.4-13.1)	11.7 (10.4-12.7)	11.9 (11.2-12.7)	12.9 (12.4-13.4)	<0.001
Hematocrit (%)	37.1 (33.9-40.0)	33.8 (30.1-36.6)	35.9 (33.3-37.8)	39.5 (37.4-41.2)	<0.001
Platelet (x10^3/uL)	269.0 (224.0-318.2)	281.0 (226.0-338.5)	259.0 (223.0-304.0)	274.0 (224.0-318.0)	0.167
Lymphocyte (%)	30.8 (23.7-36.9)	23.6 (18.5-30.1)	29.2 (22.7-35.4)	34.6 (29.2-39.0)	<0.001
Neutrophil (%)	60.8 (54.0-69.0)	69.9 (60.0-74.4)	62.6 (56.0-70.3)	57.9 (52.1-63.3)	<0.001
NLR	2.0 (1.5-3.0)	3.1 (2.0-4.2)	2.1 (1.6-3.1)	1.7 (1.3-2.2)	<0.001
SII	541.3 (370.9-861.5)	816.0 (512.3-1302.6)	573.6 (389.5-905.7)	446.1 (336.0-638.4)	<0.001

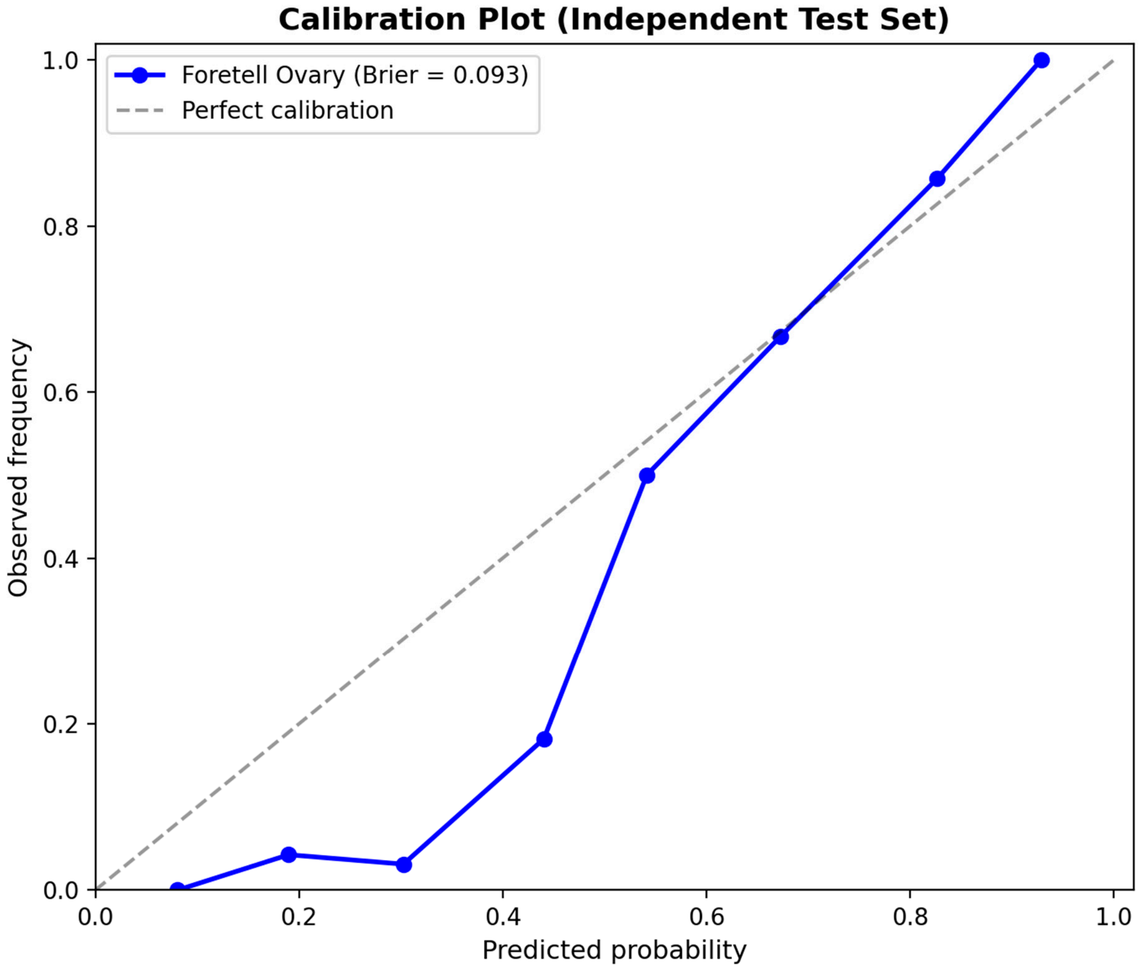

Figure S1. Calibration plot comparing predicted probabilities with observed ovarian-cancer frequencies in the independent per-protocol test set (n = 169; ovarian cancer, n = 24), using eight equal-width probability bins. The dashed diagonal line indicates perfect calibration. The Brier score was 0.093.

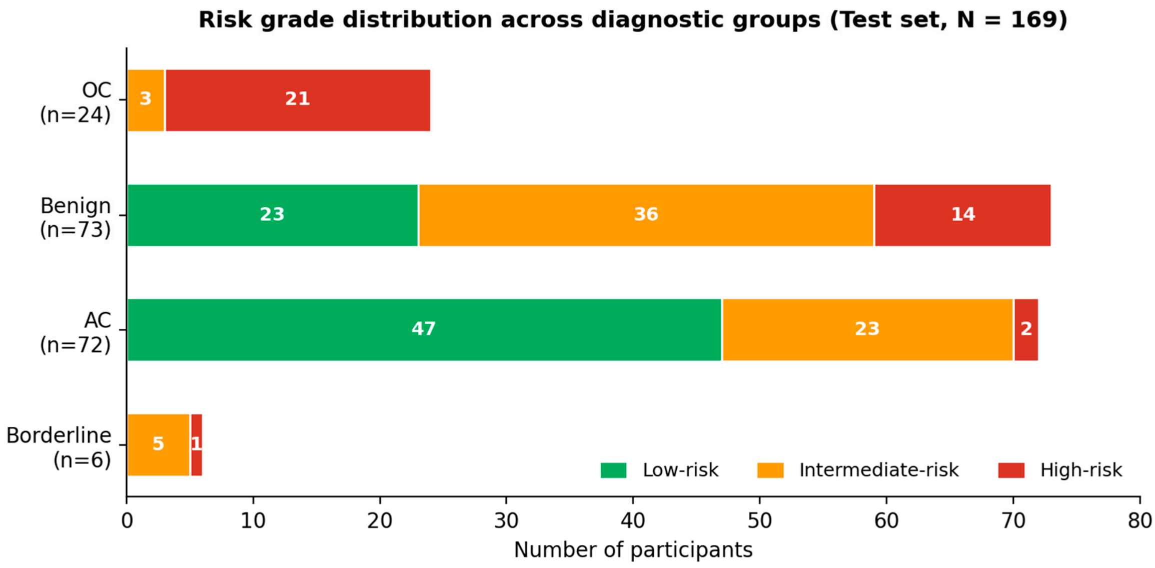

Figure S2a. Three-tier risk grade distribution across diagnostic groups in the independent test set (n = 169). Borderline ovarian tumors (n = 6) are shown separately as a reference subgroup.

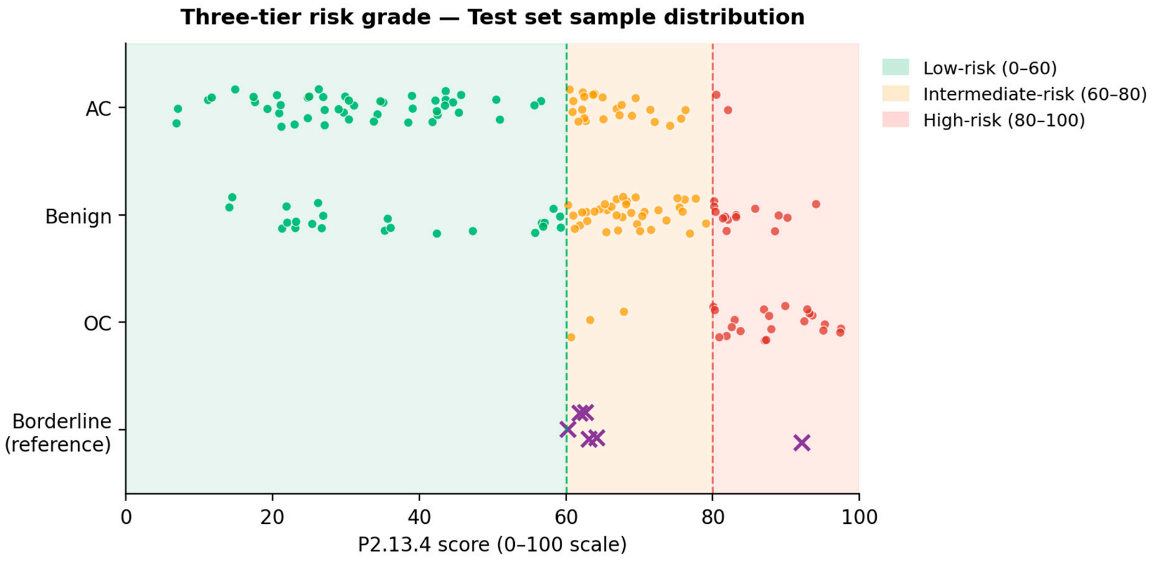

Figure S2b. Test-set sample distribution by Foretell Ovary score (0–100 point scale). Each dot represents one participant; background zones indicate risk grades: low-risk (0–60), intermediate-risk (60–80), high-risk (80–100). Borderline ovarian tumors are shown as cross markers on a separate row.

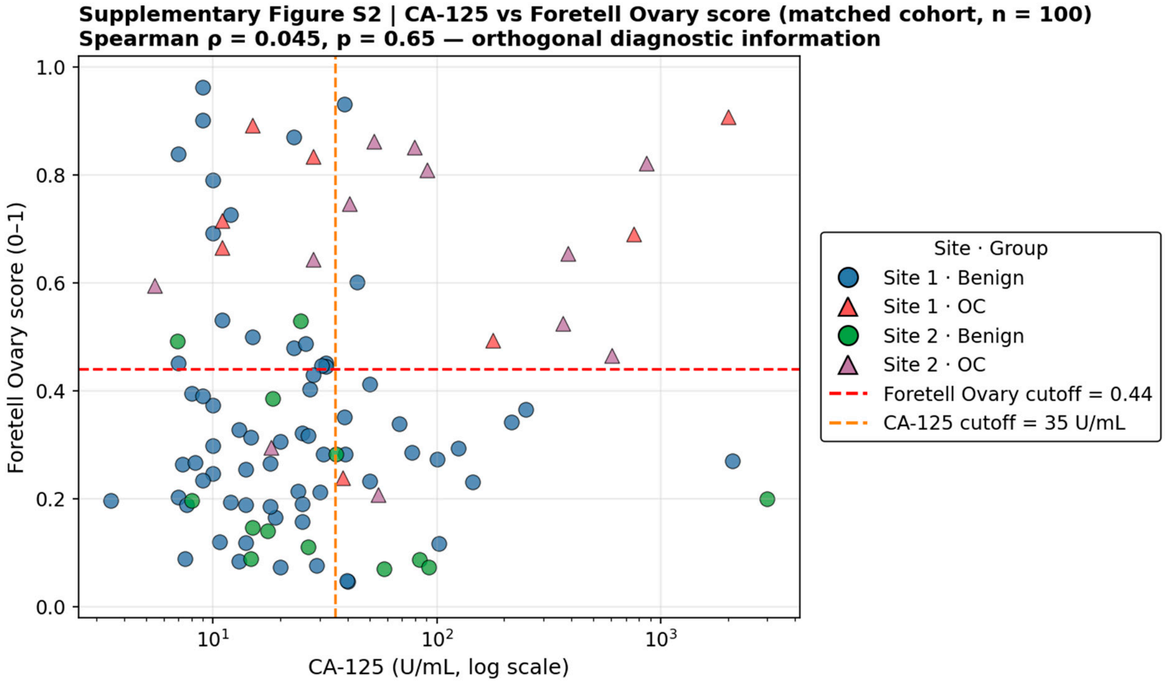

Figure S3. Scatter plot of CA-125 (U/mL, log scale) versus Foretell Ovary score (0–1) for each participant in the matched per-protocol cohort (n = 100; ovarian cancer 20, benign 80). Points are stratified by both colour (Site 1 in blue/red, Site 2 in green/purple) and marker shape (Benign as circles, ovarian cancer as triangles), so that each of the four Site × Group categories is visually distinguishable. Dashed reference lines indicate the CA-125 clinical cutoff (35 U/mL) and the Foretell Ovary classification cutoff (0.44). Spearman ρ = 0.045, p = 0.65, indicating that CA-125 and Foretell Ovary capture orthogonal diagnostic information.

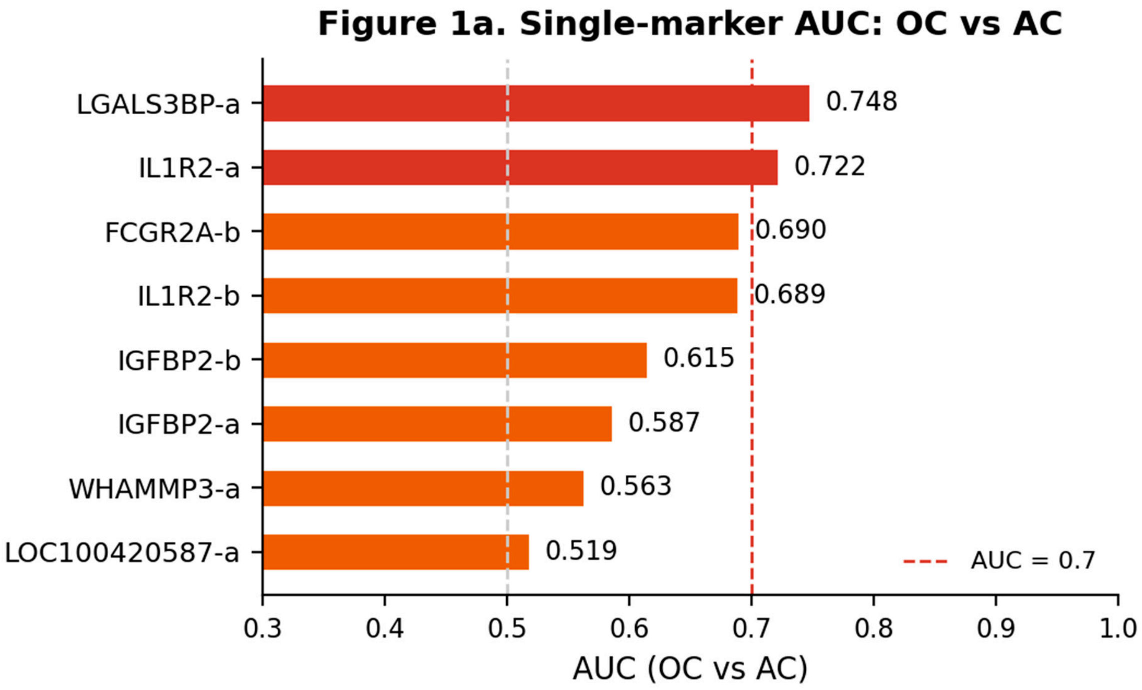

Figure S4a. Single-marker AUC summary for ovarian cancer versus asymptomatic control. Test set (ovarian cancer n = 24, asymptomatic control n = 72). Dashed gray line indicates chance level (AUC = 0.5); dashed red line indicates AUC = 0.7. Bars at or above 0.7 are highlighted.

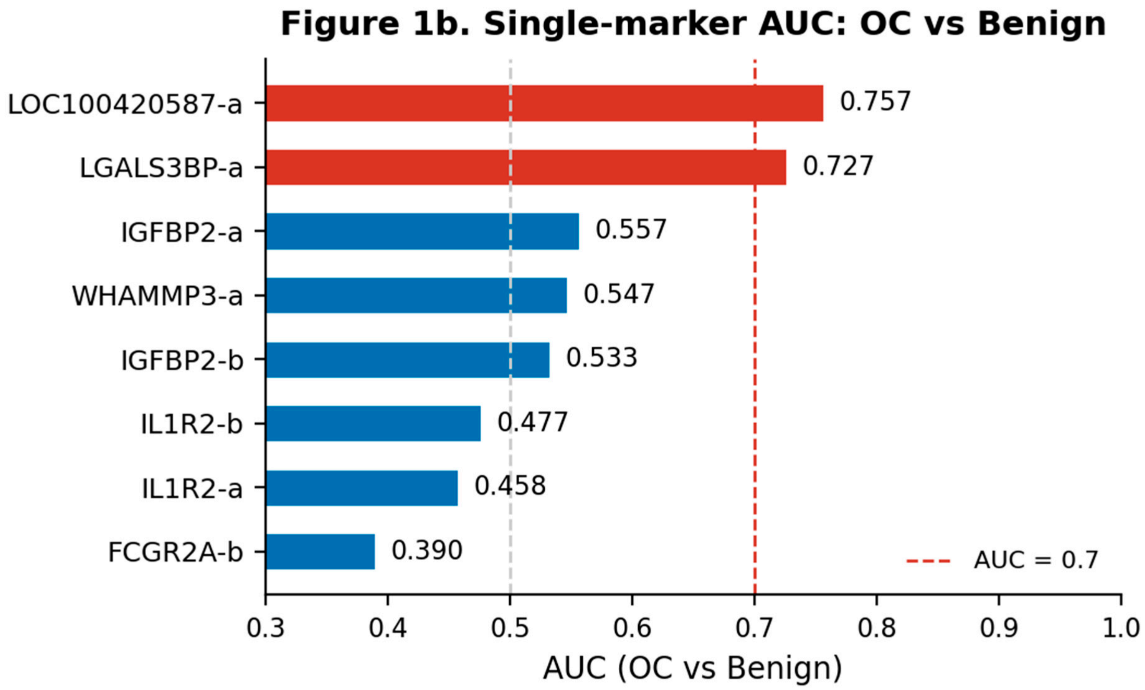

Figure S4b. Single-marker AUC summary for ovarian cancer versus benign gynecological conditions. Test set (ovarian cancer n = 24, benign n = 73).

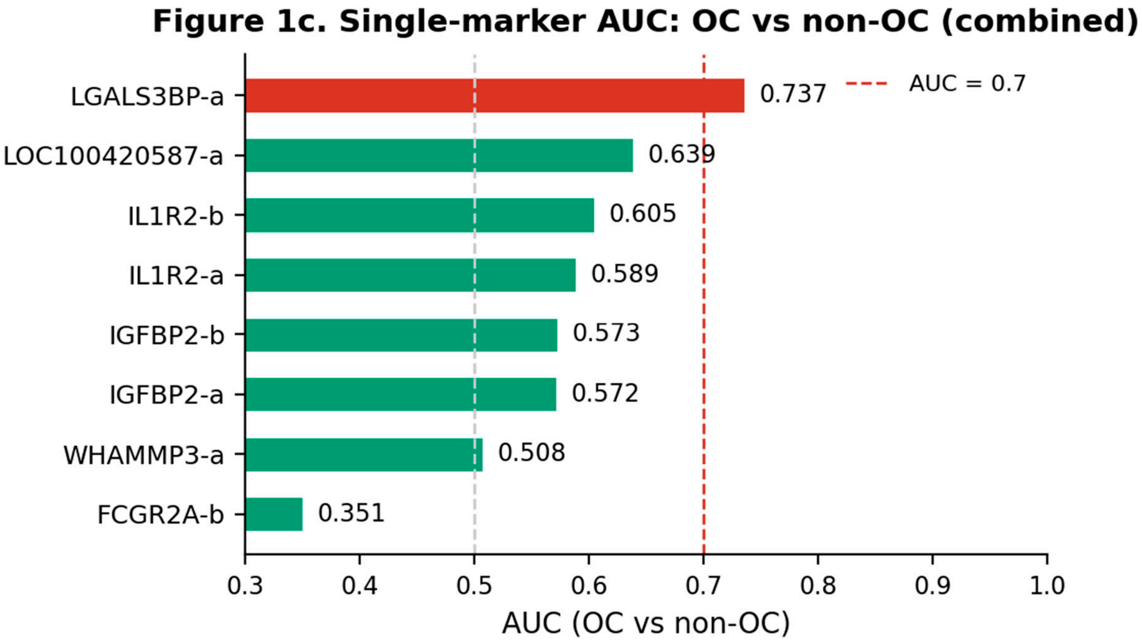

Figure S4c. Single-marker AUC summary for ovarian cancer versus combined non-ovarian-cancer comparators (asymptomatic control plus benign). Test set (ovarian cancer n = 24, non-ovarian-cancer n = 145).

S3. Robustness to pre-analytical confounders

To assess the robustness of Foretell Ovary against potential pre-analytical confounders, two complementary analyses were performed on the frozen Foretell Ovary model. First, a screening-failure cohort of 22 participants was identified across enrollment sites as having peri-sampling pharmacologic exposure (n = 10; non-steroidal anti-inflammatory drugs 3, hormonal agents 3, chemotherapy 2, antiplatelet 1, thyroid medication 1) or peri-sampling imaging (n = 12; computed tomography 5, positron-emission tomography/computed tomography 3, coronary-pulmonary angiography 3, magnetic-resonance imaging 1). Second, the relationship between platelet-rich-plasma quality-control markers (white-blood-cell and red-blood-cell contamination), ACTB cycle threshold, and model score was examined at univariate and multivariable level (n = 453 per-protocol for platelet-rich-plasma analyses; n = 436 intent-to-test for ACTB analyses).

Test-set performance was evaluated across five screening-failure-exclusion scenarios: Scenario A (all test samples, n = 175; ovarian cancer 24; reference analysis prior to the ACTB cycle threshold ≤ 28 per-protocol rule), Scenario B (all screening-failures excluded, n = 163; ovarian cancer 19), Scenario C (pharmacologic screening-failures only excluded, n = 167; ovarian cancer 22), Scenario D (imaging screening-failures only excluded, n = 171; ovarian cancer 21), and Scenario E (retraining the ensemble after removing the 10 training-set screening-failure cases and re-evaluating on Scenario B, n = 163; ovarian cancer 19). AUC was 0.916, 0.904, and 0.898 for Scenarios A, B, and E, respectively, and 0.914 and 0.907 for Scenarios C and D (all within 0.011 across progressive exclusion). Sensitivity decreased from 87.5% to 84.2%, while specificities against asymptomatic controls (98.6%) and benign gynecological conditions (82.3%) remained unchanged across all three datasets. These results indicate that screening confounders had negligible influence on overall model performance. When the per-protocol ACTB cycle threshold ≤ 28 rule is additionally applied (the standard used in the main manuscript), the same qualitative pattern holds with AUC 0.923 (full per-protocol test set n = 169) and a further marginal reduction in Scenarios B and C.

To further characterize the relationship between screening confounders and model errors, the 22 screening-failure-flagged samples were cross-referenced against all misclassified cases in the full test set under Scenario A (false positive 17, false negative 3). No screening-failure sample contributed to either false positive or false negative in the primary analysis, indicating complete non-overlap between screening-failure status and model error (Table S15). Separately, univariate correlation of model score with platelet-rich-plasma white-blood-cell contamination showed a moderate positive association (Pearson r = 0.307, |r| > 0.2 WARNING threshold); multivariable regression adjusting for group (ovarian cancer / benign / asymptomatic control) eliminated this association (p = 0.14) while identifying platelet-rich-plasma red-blood-cell contamination as an independent small negative contributor (coefficient −1.63, p = 2×10^{-4}). Marker-level analysis flagged IL1R2-a (r = −0.27), IL1R2-b (r = −0.20), and FCGR2A-b (r = −0.21). The overall risk judgment is WARNING, indicating that laboratory quality-control monitoring is required but clinical use is supported. Feature-level Mann–Whitney U tests comparing confounder-flagged versus unflagged samples yielded no significant differences across all 16 RNA features or 56 complete-blood-count-derived features after Benjamini–Hochberg false-discovery-rate correction. Principal component analysis of the full feature space confirmed that the first principal component correlated significantly with cancer group label (p = 0.0001) but not with medication status (p = 0.576) or imaging

status (p = 0.401), demonstrating that cancer-related signal dominates the feature space independently of pre-analytical confounders.

Table S14. Model performance across screening-failure-exclusion scenarios.

Metric	Scenario A (Reference, n = 175)	Scenario B (All SF excluded, n = 163)	Scenario E (Retrain + SF excluded, n = 163)
**Ovarian cancer cases (n)**	24	19	19
**AUC**	0.916	0.904	0.898
**Sensitivity**	87.5%	84.2%	84.2%
**Specificity (overall)**	88.7%	88.2%	88.2%
**Balanced accuracy**	88.1%	86.2%	86.2%

Scenario A: full test set (reference, prior to ACTB-fail per-protocol exclusion). Scenario B: excludes all 12 screening-failure samples. Scenario E: retrains the ensemble after removing the 10 training-set screening-failure cases and re-evaluates on Scenario B. Scenarios C (pharmacologic exposure only) and D (imaging only) yielded AUC 0.914 and 0.907, respectively, and are not tabulated separately. The per-protocol analysis in the main manuscript (n = 169, ACTB cycle threshold > 28 samples excluded) yields AUC 0.923; the same screening-failure-exclusion pattern is preserved. SF, screening failure; AUC, area under the receiver operating characteristic curve.

Table S15. Overlap between misclassified cases and screening-confounder samples.

Category	N	SF-flagged (n = 22)	Non-overlap (%)
**False positives**	17	0	100.0%
**False negatives**	3	0	100.0%
**Total misclassified**	20	0	100.0%

Screening-failure samples: peri-sampling pharmacologic exposure (n = 10) or imaging exposure (n = 12), identified post hoc across all enrollment sites. Non-overlap: proportion of misclassified cases with no screening-failure flag. Misclassifications counted under Scenario A (full test set).

S4. Analytical reproducibility and repeatability

Analytical reproducibility and repeatability of Foretell Ovary were evaluated under the Foretell Ovary scoring model across four hierarchical levels of pre-analytical and analytical variation: (i) intra-run PCR repeatability (same complementary-DNA template, same plate, technical replicates), (ii) complementary-DNA-synthesis repeatability (independent reverse-transcription reactions from the same RNA preparation), (iii) aliquot reproducibility (independent platelet-RNA aliquots derived from the same blood draw), and (iv) inter-draw reproducibility (separate phlebotomy events from the same subject sampled 1–6 months apart). All scores reported reflect the Foretell Ovary scoring model. Binary positive/negative concordance is taken against the 0.44 high cutoff.

S4.1 Intra-run PCR repeatability

Intra-run PCR repeatability was evaluated across 8 complementary-DNA samples drawn from two subjects, with 2–3 technical PCR replicates per complementary DNA (22 replicates total). Per-complementary-DNA score coefficient of variation ranged from 0.89% to 7.96% (mean 3.78%, maximum 7.96%). Binary positive/negative concordance against the 0.44 high cutoffwas 100.0% across all replicates.

Table S16. Intra-run PCR repeatability (same complementary DNA, same plate).

Subject	complementary DNA	n PCR replicates	Score mean ± SD	Score CV (%)
**1**	cDNA-1	2	0.265 ± 0.006	2.21
**1**	cDNA-2	3	0.195 ± 0.003	1.60
**1**	cDNA-3	3	0.222 ± 0.006	2.73
**1**	cDNA-4	2	0.229 ± 0.002	0.89
**1**	cDNA-5	3	0.207 ± 0.014	6.81
**2**	cDNA-2	3	0.292 ± 0.017	5.89
**2**	cDNA-3	3	0.290 ± 0.023	7.96
**2**	cDNA-5	3	0.309 ± 0.007	2.19

Per-complementary-DNA summary across 2–3 PCR technical replicates. Binary positive/negative concordance against the 0.44 high cutoffwas 100.0% for every complementary DNA.

S4.2 Complementary-DNA-synthesis repeatability

Complementary-DNA-synthesis repeatability was evaluated using five independent reverse-transcription reactions per subject from the same RNA preparation. Score coefficient of variation across all replicates per subject was mean 9.60%, maximum 10.94%. Binary concordance against the 0.44 high cutoff was 100.0% in both subjects.

Table S17. Complementary-DNA-synthesis repeatability (5 independent reverse transcriptions per subject from the same RNA).

Subject	n cDNA preps	n PCR replicates	Score mean ± SD	Score CV (%)
**1**	5	13	0.220 ± 0.024	10.94
**2**	5	11	0.305 ± 0.025	8.27

S4.3 Aliquot reproducibility

Aliquot reproducibility was evaluated in two healthy volunteers, each contributing four independent platelet-RNA aliquots derived from the same blood draw. Score coefficient of variation was mean 34.50%, maximum 36.90%; all eight replicates remained well below the 0.44 high cutoff (range 0.026–0.112). The elevated relative coefficient of variation reflects amplification of relative variation at the very low absolute scores observed in healthy volunteers rather than biological or analytical instability. Binary concordancewas 100.0%.

Table S18. Aliquot reproducibility (independent platelet-RNA aliquots from the same blood draw).

Subject	Draw date	n aliquots	Score mean ± SD	Score CV (%)
**A**	2025-02-26	4	0.050 ± 0.019	36.90
**B**	2025-02-26	4	0.078 ± 0.025	32.10

S4.4 Inter-draw reproducibility

Inter-draw reproducibility was evaluated in three healthy volunteers sampled at three independent phlebotomy events each (1–6 month intervals). Score coefficient of variation across draws was mean 21.28%, maximum 27.49%; score-level absolute standard deviation remained small (≤ 0.046), and no replicate ever crossed the 0.44 high cutoff. Binary concordance was 100.0%.

Table S19. Inter-draw reproducibility in healthy volunteers (1–6 month intervals).

Subject	n dates	n total draws	Score range	Score mean ± SD	Score CV (%)
**A**	3	6	0.047–0.077	0.058 ± 0.013	23.15
**B**	3	6	0.052–0.078	0.068 ± 0.009	13.22
**C**	3	3	0.114–0.226	0.167 ± 0.046	27.49

Summary

Across all four levels of pre-analytical and analytical variation (intra-run PCR, complementary-DNA synthesis, aliquot, and inter-draw), binary positive/negative classification against the 0.44 high cutoff was concordantin 100.0% of replicates and subjects. Mean score coefficients of variation were 3.78% for intra-run PCR, 9.60% for complementary-DNA-synthesis, 34.50% for aliquot, and 21.28% for inter-draw reproducibility. The elevated aliquot and inter-draw values reflect amplification of relative variation at the very low absolute scores observed in healthy volunteers (subject-mean scores 0.05–0.17) rather than biological or analytical instability; score-level absolute standard deviations remained small (≤ 0.046) and no replicate ever crossed the 0.44 high cutoff. These data support the analytical robustness of the binary Foretell Ovary output for clinical deployment.
